# Biliary Disease in a Tertiary Care Hospital: A Review of Clinical and Radiological Burden

**DOI:** 10.7759/cureus.52927

**Published:** 2024-01-25

**Authors:** Rawan A Mahdi, Ali T Naseeb, Mohammad W Almoataz, Dalal R Hubail, Yusuf S Alsaffar

**Affiliations:** 1 General Practice, Royal College of Surgeons in Ireland-Bahrain, Manama, BHR; 2 Internal Medicine, Salmaniya Medical Complex, Manama, BHR; 3 General Surgery, Salmaniya Medical Complex, Manama, BHR

**Keywords:** hospital burden, cholelithiasis, biliary pancreatitis, cholecystitis, ultrasound imaging

## Abstract

Introduction

Gallbladder disease accounts for a significant percentage of surgical admissions per year. A review of these cases was done to assess their hospital impact with an evaluation of the efficacy of radiological modalities in terms of evaluation, ideal use, and clinical application. Therefore, this study aims to review the demographics of the disease, the diagnostic yield of radiological modalities, and the overall outcome in regards to the hospital policies and medical services provided in hopes of achieving suitable clinical pathways, increasing the efficiency of gallbladder disease assessment, and limiting unwarranted investigations.

Methods

This is a single-center, retrospective study that included all the surgical emergency admissions from January 1st to December 31st 2018, in the Salmaniya Medical Complex, Kingdom of Bahrain. A total sample of 163 emergency admissions (cases) was selected from those aged 14 and older with documented biliary stones or biliary-related disease. A review of radiological modalities for diagnosis included plain radiographs (AXR, CXR), US abdomen, CT scans, and MRCP/MRI, which were then correlated with histopathological findings confirming the presence of gallstone disease. In addition to evaluating readmissions and emergency visits in terms of hospital burden.

Results

One hundred and sixty-three (10.44%) of 1,562 surgical admission cases in 2018 were diagnosed with biliary tree disease (76 males, 87 females). A total of 419 different radiological investigations were requested in 161 of the cases evaluated: 53.7% of plain radiographs (AXR, CXR), 33.2% of US abdomen, 11.9% of CT scan, and 1.2% of MRCP/MRI. Ultrasound showed a sensitivity of 48.72% and a specificity of 100%, while CT scan sensitivity was 57.14% and a specificity of 100% when it came to detecting gallstones and gallbladder-related disease. Plain radiographs add no direct benefit to diagnosing biliary disease.

Conclusion

Gallbladder disease is very prevalent with a wide array of disease entities, requiring radiological assistance in diagnosis. Ultrasound is the ideal modality for the diagnosis of biliary disease due to its ease of use and availability; it has high sensitivity and specificity, and it can be complemented by other modalities such as CT scans and MRCP/MRI when it comes to assessing for complications. On the other hand, plain radiographs have no significant value in the detection of gallbladder-related disease, and their utilization should be limited to emergency cases with high clinical suspicion.

## Introduction

Biliary disease, one of the most common and frequently encountered digestive disorders, ranking in the top 10 highest surgical admissions, has been noted to be on the rise in recent years [1-3]. Though it often remains dormant and asymptomatic in 80% of the population, the remaining 20% may become symptomatic, presenting with recurrent emergency visits, hospitalization, and a subsequent need for surgical intervention [1,4]. This entity covers a wide spectrum of pathological processes, and proper assessment is essential in identifying and providing the optimal treatment.

Radiological investigations complement the clinical diagnosis process and aid in regard to patients’ management as per their active pathology. Furthermore, it provides proper identification of diseases that warrant hospitalization or can be managed on an outpatient basis. Radiological modalities have been evolving and changing over the years, with new technologies replacing previously used methods and practices. 

The objective of this study is to review the surgical admissions and the radiological assessments to observe the burden of biliary disease in our facility. In addition, highlighting the ideal radiological modalities utilized and their diagnostic yield will help optimize clinical pathways and hospital policies.

## Materials and methods

Study design and participants**

This is a single-center, retrospective study that included all the surgical emergency (ER) admissions between the 1st of January and the 31st of December 2018 in Salmaniya Medical Complex, one of the main public tertiary care hospitals in the Kingdom of Bahrain.

Initial data provided by the admission bureau showed a total of 1,546 admission episodes under the surgical department during that year. Upon closer inspection case by case for biliary disease, an addition of 16 hospital admission episodes were noted to be missing and were added manually to conclude with a total of 1,562 cases.

The study highlighted a range of radiological modalities, which included simple abdominal and chest radiographs (AXR and CXR) and more sophisticated tools such as ultrasonography (US), computed tomography scans (CT scans), and magnetic resonance cholangiopancreatography (MRCP/MRI). This was also contrasted against clinical diagnosis and laboratory investigations. Supplementary radiological studies were obtained from other governmental or private healthcare centers in the kingdom by the patients before their admission to our facility.

Ethical approval was submitted, reviewed, and gained for this study.

Inclusion criteria

Patients aged 14 years and older with documented biliary stones or biliary-related diseases, such as biliary pancreatitis, and surgical complications related to operative management. These cases were identified and included based on proven clinical assessment or radiological evidence confirming the presence of disease. The hospital burden was accounted for based on the number of episodes, visits, and admissions rather than the actual number of individuals admitted.

Exclusion criteria

Non-biliary disease-related cases that had no clear evidence of existing cholelithiasis or a clinical picture suggestive of gallbladder disease and associated complications were excluded. Similarly, cases of acute pancreatitis without any definitive cause of biliary stones as a primary risk factor were eliminated. Patients who visited the emergency department with mild biliary colic or cholecystitis and did not require admission were omitted from the study population. One case of ascending cholangitis was excluded, as these patients are often admitted and managed by gastroenterology in our facility. Patients admitted with abdominal pain for observation with an unspecified cause, in addition to those who presented with a clinical picture of gallstones and yet signed against medical advice or absconded during their emergency department assessment, were also excluded. Patients admitted under the care of non-surgical specialties with concomitant gallbladder disease or who developed evidence of gallstone complications during their hospital stay were not factored in.

Sample size

The initial sample selected of 184 cases included biliary-related diagnoses as well as non-biliary-related surgical admissions with other diagnoses who had documented cholelithiasis or obesity as comorbidities and risk factors. A total of 21 cases were excluded to settle on a final population of 163 cases for this study.

Data collection

The data was collected from both the electronic database and the patients’ medical records department. The gathered information featured the demographics of the patients, comorbidities, length of hospital stay, various radiological modalities requested, and the primary treatment of choice provided, in addition to recurrent emergency visits within a one-year time frame from the index admission. The radiological diagnostic yield was determined based on the radiological assessment and reports provided. These were designated interpretations as follows (Table 1).

**Table 1:** Diagnostic yield categories

Category	Definition
Diagnostic Positive	A clear finding that indicates and confirms the presence of a pathology as well as a specific diagnosis.
Diagnostic Negative	Negative radiological finding excluding presence of other differential diagnosis, with subsequent confirmation of a diagnosis based on clinical assessment.
Non-Diagnostic Positive	A positive finding was identified on the imaging but did not aid in reaching a final diagnosis.
Non-Diagnostic Negative	Negative findings on imaging and did not aid in excluding other diseases.

Data analysis

Google Documents (Docs: Spreadsheet) and Microsoft Excel were the main methods utilized to compute and analyze data. The acquisition of patients’ demographic data was obtained through a list provided by the hospital admissions bureau. A sample selection of 163 cases was inspected for clinical diagnosis and spectrum of biliary disease, the sum of radiological assessment tools and diagnostic yield, and recurrent case evaluation to determine the overall patient and hospital burden. 

## Results

Data demographics

The general surgery emergency admissions in the year 2018 in patients aged 14 and above totaled 1,562 cases. Of these, 163 (10.44%) were cases of biliary tree disease (76 males, 87 females). The final study sample (n=163) included a total of 139 individuals after taking into account repeated admissions. The youngest recorded age was 16 years old, while the eldest was 101 years old at the time of admission and lived to the age of 106. The standard deviation of the study was 16.4, with a mean age and confidence interval of 95% around 44.5 ±2.52 years.

**Figure 1:**
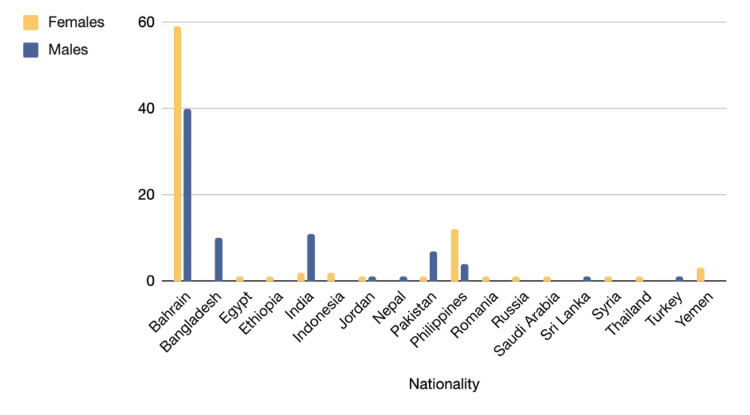
General demographics: Gender and nationality distribution n=163 (10.44%) of the starting 1,562 data which were divided into gender (76 males [46.6%] and 87 females [53.4%]) and nationality. Highest values in following countries: Bahrain (n=99, 60.7%), Philippines (n=16, 9.8%), India (n=13, 8%), Bangladesh (n=10, 6.1%), Pakistan (n=8, 5%) and others (n=17,  10.4%), respectively

The collective number of non-nationals (expats) was noted at 63 cases, with the highest values found in the following countries of origin: the Philippines (n=16), India (n=13), Bangladesh (n=10), Pakistan (n=8), respectively. Additional details can be found in Figure 1.

**Figure 2:**
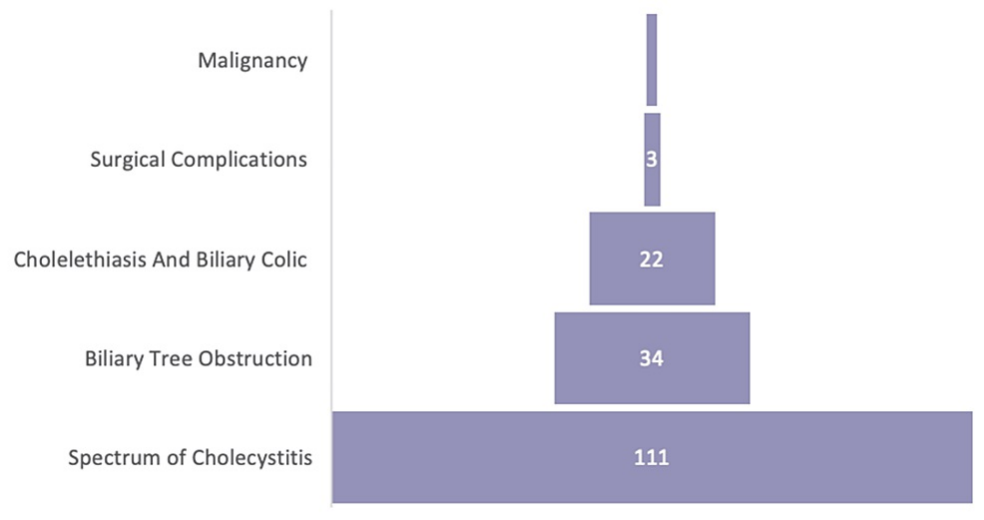
Diagnosis trends based on categories Total (n=172) different diagnoses. Spectrum of cholecystitis (n=111, 64.5%), biliary tree obstruction (n=34, 19.8%), cholelethiais and biliary colic (n=22, 12.8%), surgical complications (n=3, 1.7%), and malignancy (n=2, 1.2%).

**Figure 3:**
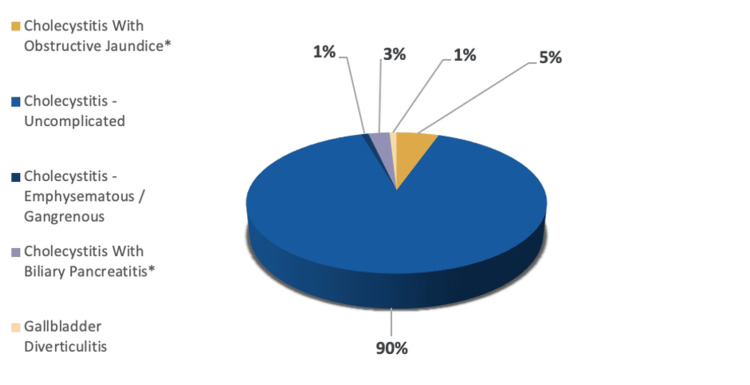
The spectrum of cholecystitis disease *Total number of diagnoses adjusted for concomitant pathology The spectrum of cholecystitis (n=111) is distributed into subtypes: Cholecystitis uncomplicated (n=100, 90%), cholecystitis with obstructive jaundice (n=6, 5%), cholecystitis with biliary pancreatitis (n=3, 3%), cholecystitis with emphysematous/gangrenous (n=1, 1%), gallbladder diverticulitis (n=1, 1%)

The collected data (n=163) was analyzed and grouped into categories for representation purposes; however, adjustments were made taking into consideration concomitant conditions (n=172). These included the spectrum of cholecystitis (n=111), biliary tree obstruction (n=34), cholelethiais and biliary colic (n=22), surgical complications (n=3) and malignancy (n=2). Patients with two separate diagnoses were accounted for in their respective categories. For example, cases admitted for biliary pancreatitis with cholecystitis were accounted for twice in both the cholecystitis and the biliary tree groups despite being the same patient (Figures 2, 3).

Radiological statistics

**Figure 4:**
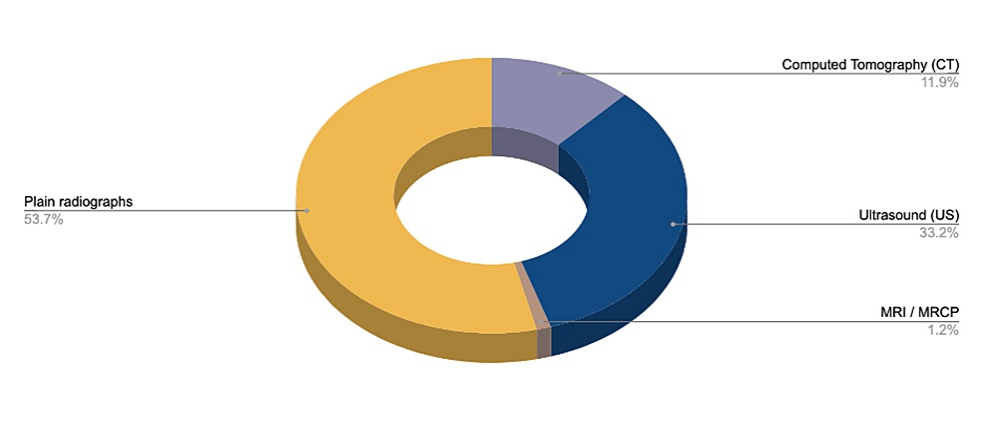
Imaging modalities % Modalities supporting the diagnosis of biliary disease studied in our population (n=163) in our facility. Plain radiographs (n=225, 53.7%), US (n=139, 33.2%), CT scan (n=50, 11.9%), MRCP (n=5,  1.2%)

Modalities supporting the diagnosis of biliary disease studied in our population (n=163) included: plain radiographs, US abdomen, CT scan, MRCP/MRI, as well as radiological reports provided by the patient from external hospitals or facilities. Plain radiographs (AXR and CXR) accounted for 53.7%, CT scans were 11.9%, MRCP were 1.2%, and lastly, the gold-standard modality of choice for biliary tree assessment, US, at 33.2%. Additionally, 18 cases that had ultrasounds done in a private hospital were identified; however, they were not factored into the total percentage of imaging done locally (Figure 4).

The data in question was reviewed to quantify the role radiological assessment played in the clinical diagnosis of biliary disease, and the results presented themselves as follows: a total of 419 different radiological investigations were requested in 161 of the cases evaluated. A collective of 225 plain radiographs (abdominal X-ray [AXR] and chest X-ray [CXR]) were done (120 males and 105 females), in addition to some cases having a form of advanced imaging with either an US or CT scan at least requested (Table 2).

**Table 2:** Overview of radiological investigations Plain radiograph: CXR (chest X-ray)/AXR (abdominal X-ray) M: Male, F: Female. 161 cases (98.8%) of the n=163 population were divided into males and females and total 419 radiological investigations were done in our facility to investigate biliary disease. Plain radiographs (n=225, 53.7%), male (n=120, 53.3%) female (n=105, 46.7%). US (n=139, 33.2%), male (n=64, 46%), female (n=75, 54%). CT scan (n=50, 11.9%), male (n=34,  68%), female (n=16, 32%). MRCP (n=5, 1.2%), male (n=2, 40%), female (n=3, 60%)

Modality: Total number of cases with radiological assessment = 161/163	M	F	Total
Plain radiographs (CXR/AXR)	120	105	225
Ultrasound (US)	64	75	139
Computed Tomography (CT)	34	16	50
Magnetic resonance cholangiopancreatography (MRCP)	2	3	5
Total Imaging performed	220	199	419

Plain radiographs comprised a large percentage (53.7%) of the imaging modalities requested, with a total number of 225 divided between 108 CXR (58 males and 50 females) and 117 AXR (62 males and 55 females). Diagnostic yield was classified into four categories, as mentioned in the Methods section, to assess the radiological role in disease detection or exclusion.

Reviewed plain radiographs showed a majority of non-diagnostic negative investigations performed with 96 CXRs and 109 AXRs. The second highest category was 17 cases in the non-diagnostic positive category, followed by three cases that were diagnostic negative. Diagnostic positive cases accounted for 0 images requested (Table 3).

**Table 3:** Plain radiographs have a diagnostic yield The number of cases with plain radiographs is 138 (84.6%) out of 163 sample populations. Diagnostic positive (n=0, 0%), diagnostic negative (n=3, 1.3%), non-diagnostic positive (n=17, 7.5%), non-diagnostic negative (n=205, 91.1%) Chest X-ray Diagnostic positive: male (n=0, 0%), female (n=0, 0%) Diagnostic negative: male (n=1, 33.3%), female (n=1, 33.3%) Non-diagnostic positive: male (n=1, 5.9%), female (n=9, 53%) Non-diagnostic negative: male (n=56, 27.3%) female (n=40, 19.5%) Abdominal X-ray Diagnostic positive: male (n=0, 0%), female (n=0, 0%) Diagnostic negative: male (n=1, 33.3%), female (n=0, 0%) Non-diagnostic positive: male (n=6, 35.3%), female (n=1, 5.9%) Non-diagnostic negative: male (n=55, 26.8%), female (n=54, 26.3%)

Modality: Plain Radiographs	Plain Chest X-rays		Plain Abdominal X-rays		Total
	Male	Female	Male	Female	
Diagnostic Positive	0	0	0	0	0
Diagnostic Negative	1	1	1	0	3
Non-Diagnostic Positive	1	9	6	1	17
Non-Diagnostic Negative	56	40	55	54	205
Total	58	50	62	55	225

Ultrasound is the principal modality of interest being evaluated as it is the most recommended tool in the assessment of biliary disease, and in our study, it was noted to be performed on 139 patients out of the sample population. Radiology analysis with the presence of findings showed that 124 cases featured diagnostic-positive results in contrast to six non-diagnostic-positive reports. On the other hand, the absence of findings was noted in seven diagnostic-negative studies and two non-diagnostic-negative studies. The gender distribution of ultrasound diagnostic yields can be found in Table 4.

**Table 4:** Ultrasound diagnostic yield * 1 Saudi female (GCC citizen) is accounted for as national in the diagnostic positive  (refer to Appendix) Number of cases with ultrasound: 139 out of 163 (85.3%). Diagnostic positive (n=124,  89.2%), diagnostic negative (n=7, 5%), non-diagnostic positive (n=6, 4.3%), non-diagnostic negative (n=2, 1.4%) National Diagnostic positive: male (n=28, 22.6%), female (n=44, 35.8%) Diagnostic negative: male (n=0, 0%), female (n=5, 71.4%) Non-diagnostic positive: male (n=3, 50%), female (n=0, 0%) Non-diagnostic negative: male (n=0, 0%), female (n=0, 0%) Non-National Diagnostic positive: male (n=29, 23.4%), female (n=23, 18.5%) Diagnostic negative: male (n=1, 14.3%), female (n=1, 14.3%) Non-diagnostic positive: male (n=2, 33.3%), female (n=1, 16.7%) Non-diagnostic negative: male (n=1, 50%), female (n=1, 50%)

Ultrasound-Diagnostic Yield	National		Non-National		Total
	Male	Female*	Male	Female	
Diagnostic Positive	28	44	29	23	124
Diagnostic Negative	0	5	1	1	7
Non-Diagnostic Positive	3	0	2	1	6
Non-Diagnostic Negative	0	0	1	1	2
Total	31	49	33	26	139

**Figure 5:**
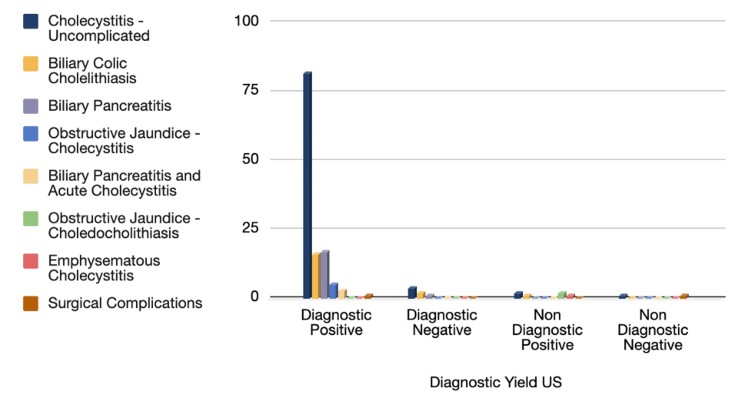
Ultrasound diagnostic yield: admitting diagnosis Number of cases with ultrasound: 139 out of 163. Cholecystitis: uncomplicated (n=89 - 64%), biliary colic: cholelithiasis (n=19, 13.7%), biliary pancreatitis (n=18, 13%), obstructive jaundice: cholecystitis (n=5, 3.6%), biliary pancreatitis and acute cholecystitis (n= 3, 2.2%), obstructive jaundice: choledocholithiasis (n=2, 1.4%), emphysematous cholecystitis (n=1, 0.7%), surgical complications (n=2, 1.4%).

Furthermore, a correlation between the admitting diagnosis and ultrasound diagnostic yield was analyzed, as seen in Figure 5, noting that the most frequent diagnoses requiring US assessment were the following: acute cholecystitis, followed by biliary colic and biliary pancreatitis, respectively.

**Figure 6:**
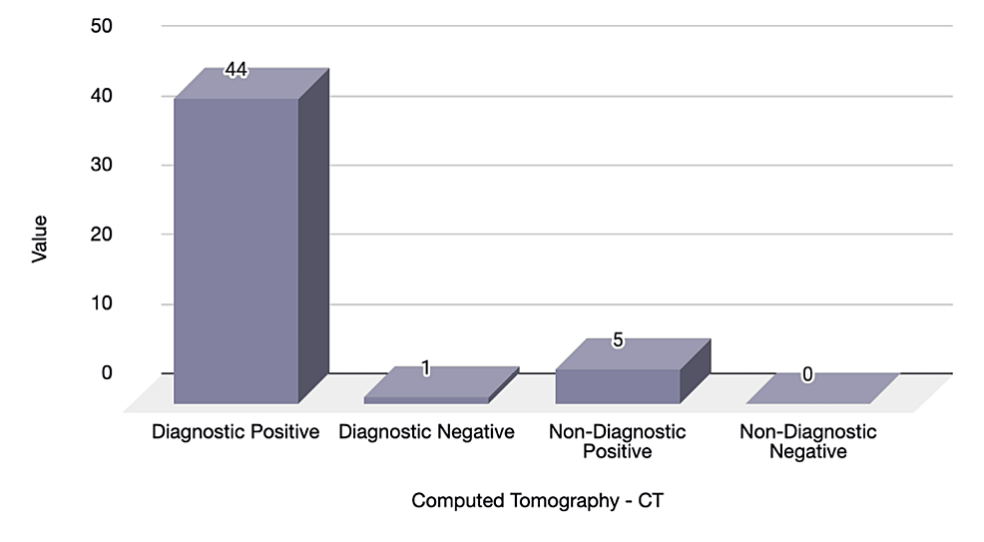
CT Abdomen diagnostic yield Number of cases with CT scan: 50 out of 163. Diagnostic positive (n=44, 88%), Diagnostic negative (n=1, 2%), non-diagnostic positive (n=5, 10%), non-diagnostic negative (n=0, 0%)

**Figure 7:**
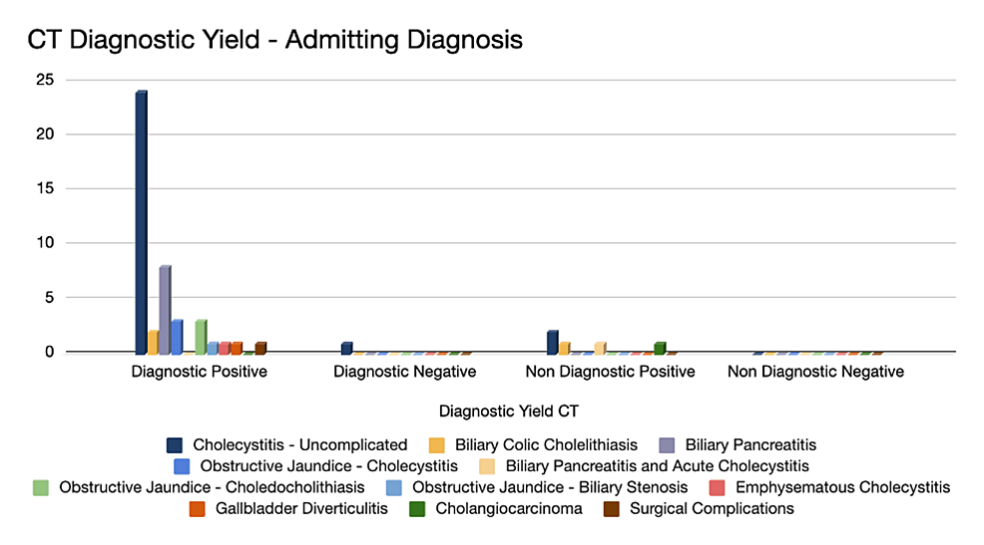
CT diagnostic yield: admitting diagnosis Number of cases with CT scan: 50 out of 163. Cholecystitis: Uncomplicated (n=27,  54%), biliary colic: cholelithiasis (n=3, 6%), biliary pancreatitis (n=8, 16%), obstructive jaundice: cholecystitis (n=3, 6%), biliary pancreatitis and acute cholecystitis (n= 1, 2%), obstructive jaundice: choledocholithiasis (n=3, 6%), obstructive jaundice: biliary stenosis (n=1, 2%), emphysematous cholecystitis (n=1, 2%), gallbladder diverticulitis (n=1, 2%), cholangiocarcinoma (n=1, 2%), surgical complications (n=1, 2%)

Collectively, 50 CT scans were done (34 males, 16 females). Out of those, 44 cases were diagnostically positive (88%), and five accounted for non-diagnostic imaging with positive findings (10%); the remaining cases were depicted as diagnostically negative (2%). The diagnostic yield is featured in Figure 6. The diagnosis of cholecystitis had the highest percentage of CT scans performed for diagnostic purposes and the identification of pathologies. Further analysis was computed against the diagnosis list (Figure 7).

**Figure 8:**
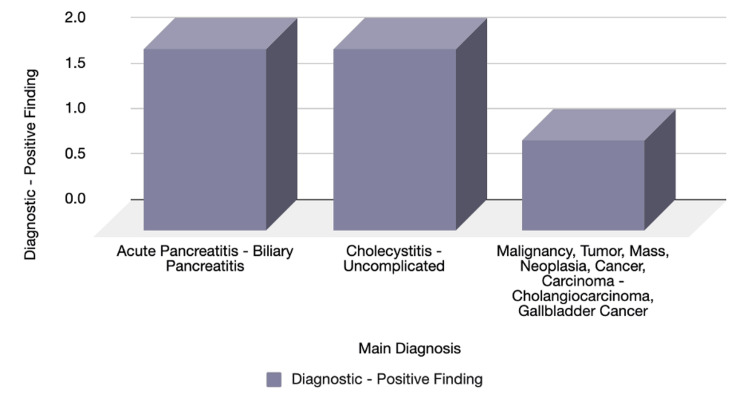
MRI diagnostic yield Number of cases with MRCP/MRI: five out of 163. Acute pancreatitis: biliary pancreatitis (n=2, 40%), cholecystitis: uncomplicated (n=2, 40%), malignancy (n=1, 20%).

Lastly, MRCP was utilized and found to be diagnostic positive in all five cases (1.2%) encountered in this study. A schematic graph demonstrates these findings with a highlighted admitting diagnosis (Figure 8).

Patients who had imaging modalities done in private hospitals and health care centers subsequently attending our facility accounted for 18 cases in this study and were all noted to be sonographic assessments. These reports were either provided upon their current admission as active complaints or presented as documentation of a prior episode that was resolved.

**Figure 9:**
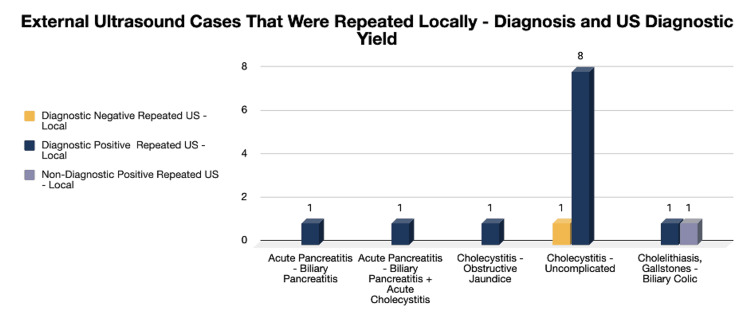
External ultrasound cases that were repeated locally: diagnosis and US diagnostic yield 14 cases out of the 163-sample population had repeated ultrasound imaging both externally (in a private hospital) and locally in our facility. Cholecystitis: uncomplicated (n=9, 64.3%), cholecystitis with obstructive jaundice (n=1, 7.14%), acute pancreatitis, biliary pancreatitis (n=1, 7.14%), acute pancreatitis, biliary pancreatitis + acute cholecystitis (n=1, 7.14%), cholelithiasis, gallstones: biliary colic (n=2, 14.3%).

Ultrasounds done externally were cross-checked with studies performed at our facility and were noted to include 14 repeated US requests, while six cases had a CT scan as a repeat or further assessment method. These locally repeated investigations were contrasted into the following diagnostic yields as per the admitting diagnosis with the highest frequency, which was noted to have a diagnostic positive of eight cases and one diagnostic negative in the cholecystitis domain (Figure 9).

A summary of diagnostic yields is provided in Table 5, highlighting plain radiographs as the most frequent modality requested with the poorest diagnostic yield, as opposed to ultrasound, which has the highest diagnostic power in detecting biliary disease despite being the second most frequent modality. Ultrasound is considered the first-line and gold-standard modality of assessment, following a 100% diagnostic power noted in the less frequently requested MRI studies.

**Table 5:** Imaging modality types and diagnostic yield overview CXR: chest X-ray, AXR: abdominal X-ray, US: ultrasound, CT: computed tomography scan, MRCP/MRI: magnetic resonance cholangiopancreatography/magnetic resonance imaging Number of cases who had radiological investigations (plain radiographs, US, CT scan) = 161 out of 163 (98.8%). Diagnostic positive (n=173, 41.3%), diagnostic negative (n=11,  2.6%), non-diagnostic positive (n=28, 6.7%), non-diagnostic negative (n=207, 49.4%) Diagnostic positive: CXR (n=0, 0%), AXR (n=0, 0%), US (n=124, 72.1%), CT (n=44, 25%), MRCP/MRI (n=5, 2.9%) Diagnostic negative: CXR (n=2, 18.3%), AXR (n=1, 9%), US (n=, %) CT (n=1, 9%), MRCP/MRI (n=0, 0%) Non-diagnostic positive: CXR (n=10, 35.7%), AXR (n=7, 25%), US (n=6, 21.4%), CT (n=5,  17.8%), MRCP/MRI (n=0, 0%) Non-diagnostic negative: CXR (n=96, 46.4%), AXR (n=109, 52.6%), US (n=2, 0.96%), CT (n=0, 0%), MRCP/MRI (n=0, 0%)

Diagnostic Yield	CXR	AXR	US	CT	MRCP/MRI	Total
Diagnostic Positive	0	0	124	44	5	173
Diagnostic Negative	2	1	7	1	0	11
Non-Diagnostic Positive	10	7	6	5	0	28
Non-Diagnostic Negative	96	109	2	0	0	207
Total	108	117	139	50	5	419

Comparison of radiological and histopathological results

Out of the 163 cases studied (139 individuals), 51 patients underwent laparoscopic cholecystectomy in our facility. The role of US and CT scans can be emphasized by determining the sensitivity and specificity of their diagnostic strengths when contrasted against patients who underwent surgical intervention in the form of laparoscopic cholecystectomy, histological assessment, and the presence of stones. On follow-up review of the studied population, the following values were obtained: US showed a sensitivity of 48.72% and a specificity of 100%, while CT scan sensitivity was 57.14% and a specificity of 100%. Refer to the following tables, Tables 6, 7.

**Table 6:** Accuracy of ultrasound diagnosis N= 41 ultrasounds Out of 163 cases reviewed (139 individuals; adjusted for readmissions), 51 individuals (36.69%) underwent laparoscopic cholecystectomy in our hospital. The remainder was not accounted for as the surgery was not done and/or was not documented in our facility. Further details are in the Discussion section. Out of those 51 patients, 41 had ultrasounds done in our facility (80.39%). Positive histopathology and radiology (n= 19, 46.34%), positive histopathology and negative radiology (n=0, 0%), negative histopathology and positive radiology (n=20, 48.78%), negative histopathology and radiology (n=2, 0.88%) on ultrasound imaging.

US Diagnostic Power	Positive Histopathology	Negative Histopathology
Positive Radiology	19	20
Negative Radiology	0	2

**Table 7:** Accuracy of CT scan diagnosis N= 16 CT scans Out of the 163 cases reviewed (139 individuals adjusted for readmissions), 51 individuals (36.69%) underwent laparoscopic cholecystectomy in our hospital. The remainder was not accounted for as the surgery was not done and/or was not documented in our facility. Further details are in the Discussion section. Out of those 51 patients, 16 had a CT scan done in our facility (31.37%); however, this does not exclude them from having had a previous ultrasound. Positive histopathology and radiology (n= 8, 50%), positive histopathology and negative radiology (n=0, 0%), negative histopathology and positive radiology (n=6,  37.5%), negative histopathology and radiology (n=2, 12.5%) on CT scan imaging.

CT Diagnostic Power	Positive Histopathology	Negative Histopathology
Positive Radiology	8	6
Negative Radiology	0	2

Recurrent hospital admissions

**Figure 10:**
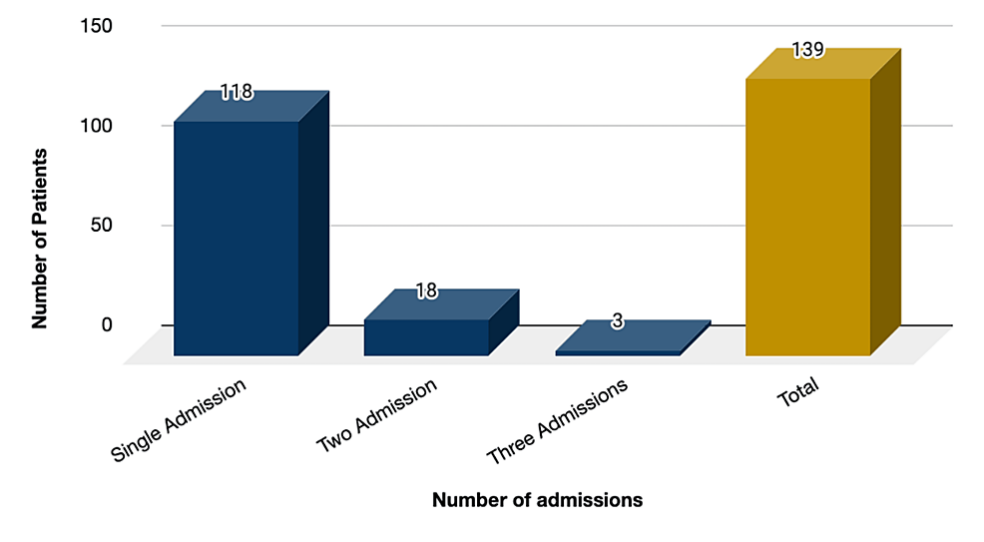
Recurrent admissions Out of the n=163 sample population, 118 had a single admission (72.39%), 18 had two admissions (11.04%), and three cases had three admissions (1.84%).

Data analysis provided that out of the 163 cases, a total of 139 patients were admitted with gallbladder disease. Of those, 118 had a single admission (72.39%), 18 had two admissions (11.04%), and three cases had three admissions (1.84%), as noted in Figure 10.

**Figure 11:**
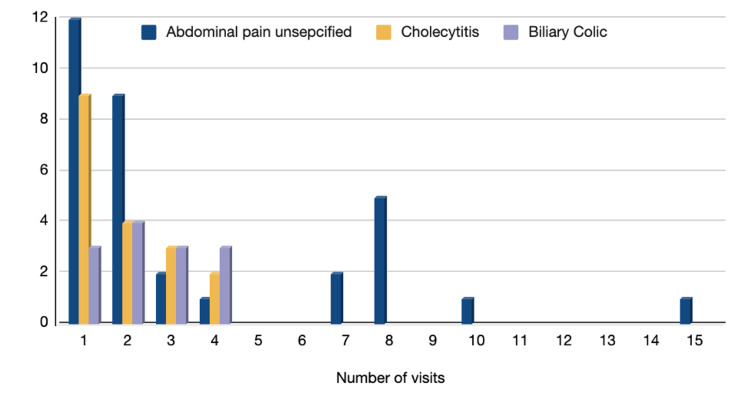
Most common diagnosis for revisits within one year Out of 139 individuals (n=163 cases, adjusted for patients with readmissions): 21 patients were readmitted more than twice within a one-year time period from the index admission. The graph shows a total of 77 emergency episodes out of n=163 cases (adjusted for patients with readmissions) and the most common cause of revisits within a one-year time period from index admission: Abdominal pain (n=33, 43%), cholecystitis (n=18,  23.4%), biliary colic (n=13, 16.8%), and others (n=13, 16.8%).

A total of 77 emergency episodes were analyzed (adjusted for patients with readmissions), and the reasons for readmissions included a recurrent episode with a similar complaint, a concomitant complication, or admission for another diagnosis from the spectrum of biliary disease. The most common causes for revisits within a one-year time frame were as follows: abdominal pain (n=33), cholecystitis (n=18), biliary colic (n=13) and the remaining isolated diagnoses were combined and labeled as others (n=13) (Figure 11). Examples of other causes included peptic ulcer disease, minimal surgical complications, and pain attributed to obstetric and gynecological complaints, to name a few; however, they were minor and dispersed cases.

## Discussion

Gallstones and biliary tree-related diseases are recognized as frequent causes of hospital visits and are considered one of the most expensive surgical gastrointestinal disorders. These spectra of pathologies are on the rise and are ranked in the top 10 reasons for surgical-related hospitalization, exerting a significant epidemiological and economic burden worldwide [1-3,5]. 

After reviewing 1,562 admissions in the year 2018 under the surgical department at our facility, this disease spectrum was noted to fill the third-highest spot, second only to acute appendicitis and skin infection-related diseases. 

Our evaluation of gallstone disease has noted right upper abdominal pain (160 out of 163 cases) to be the dominant hallmark of the disease, with associated non-specific symptoms such as nausea and vomiting. Despite this, studies describe it as a silent disease in 80% of the population, while the remaining 20% may suffer from symptoms ranging from constant severe pain to a variety of complications such as cholecystitis, cholangitis, obstructive jaundice and biliary pancreatitis [2,4]. These patients often have a 69% risk of recurrence of symptoms within a two-year time frame [4]. 

**Figure 12:**
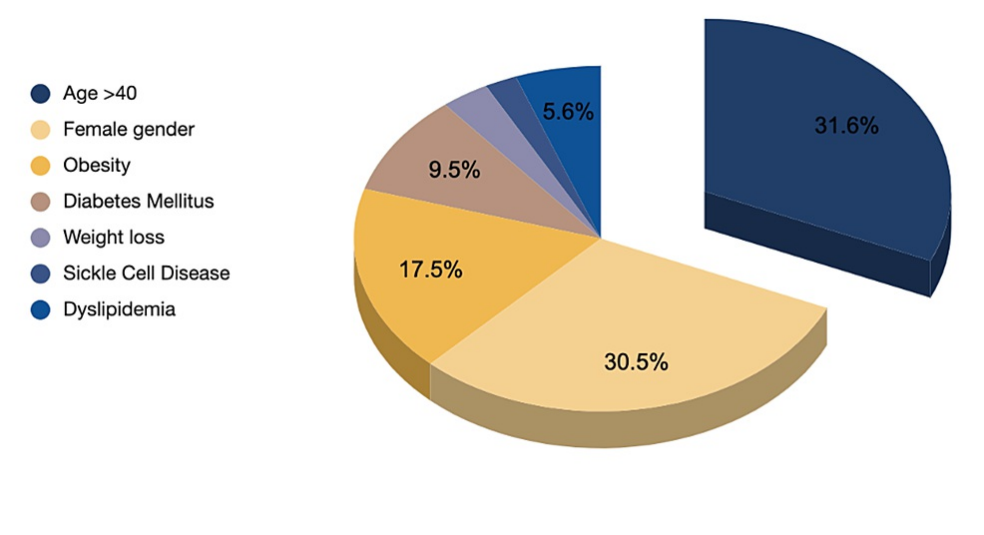
Risk factors for gallstones The comorbidities taken into account in this study included entities directly linked to biliary disease development. Their presence in all cases accounted for 139 patients (adjusted for the same individuals with recurrent visits) and was calculated at 285 comorbidities with a direct link to biliary disease development. The graph demonstrates percentages based on their frequency: Age >40 (n=90, 31.6%), female gender (n=87, 30.5%), obesity (n=50,  17.5%), diabetes mellitus (n=27, 9.5%), weight loss (n=9, 3.2%), sickle cell disease (n=6, 2.1% ), dyslipidemia (n=16, 5.6%)

Patients visiting the hospital have a long list of comorbidities, and when analyzing the risk factors that had a direct link to developing biliary disease, a total of 285 comorbidities were calculated in 139 individuals out of 163 sample cases. The risk factors for developing gallbladder disease include age (40-69 years), female sex, high parity, ethnicity, obesity or overweight, rapid weight loss (due to diet or bariatric surgery), and diabetes. When it comes to younger age groups, risk factors often occur in the setting of hemolysis or due to genetic factors [3-6]. On the same note, the highest values of risk factors in our sample population were noted to be in those aged 40 and above (31.6%), female gender (30.5%), and obesity (17.5%), respectively (Figure 12). 

Interestingly, only six cases were found to be afflicted with sickle cell disease (SCD), a prevailing genetic hemolytic disease in the Kingdom of Bahrain. This finding is striking, considering that hemoglobinopathies are common, as suggested by a previous study conducted on a population of 50,000 newborns in Bahrain that found 44.35% of the blood samples detected abnormal hemoglobin (24.2% were thalassemia cases, 18.1% showed sickle-cell trait [SCT], and 2.1% had SCD) [7]. This could be attributed to the fact that patients with hemoglobinopathies are frequently admitted under the hematology and hereditary disease specialties and were therefore excluded from our sample. 

As a disease with a wide range of presentations and underlying pathological processes (Figure 2), it is without a doubt that radiology plays an integral part in identifying diagnoses and delivering the most appropriate management and surgical intervention. Plain radiographs were first introduced to the scene by Wilhelm Roentgen, dating as far back as 1895. Since then, radiological advances have come a long way and continued to evolve further with the introduction of ultrasound-based modalities in 1950-1970 and the development of more sophisticated technology and tools used today, such as CT scans, MRIs and hepatobiliary iminodiacetic acid scans (HIDA) [5,8]. Other invasive modalities include endoscopic retrograde cholangiopancreatography (ERCP) or conventional cholangiogram. These modalities have been recorded and stored digitally in recent years.

Female gender, one of the primary risk factors for developing gallstones, is noted to have a (1.5-2: 1) female to male ratio [6,9]. Our study initially showed a F:M ratio of (1:1), but when adjusted to the F:M ratio of nationals only, it was noted to be (1.5:1.1), which is paralleled to worldwide demographics. This discrepancy was due to the population of non-nationals (expats) comprising 38.7% of the sample size (Figure 1).

In regards to the power of radiological modality as an imaging tool, it was made clear that plain radiographs played little role in the diagnosis of biliary disease, as highlighted in Table 5, with a diagnostic positive value of 0%. The number of radiographs (225) could be rationalized as an expeditious screening tool utilized by the emergency staff to rule out more sinister pathologies such as a perforated viscus or renal stones. This corresponds to 10% or less of the global detection rate of biliary stones and other findings such as porcelain gallbladder or even pneumobilia on rare occasions [10,11]. 

Ultrasound is considered the gold-standard imaging practice for the assessment of patients with abdominal pain and a clinical picture suggestive of gallbladder disease. This is due to its availability, low cost, lack of ionizing radiation, and sensitivity of 81%-86% and specificity of 83%-92% when it comes to detecting gallstones [5,12-14]. In other sources, it reported a sensitivity of 97% and a specificity of 95% [15]. Although it is operator-dependent, ultrasound is still able to provide us with several useful pieces of information, such as the presence or absence of gallstones, identifying gallbladder wall thickening, edema, and biliary sludge, ruling out non-biliary pathologies, and differential diagnosis [5].

Revision of our data reproduced results mimicking global statistics while assessing the diagnostic power of ultrasound in the spectrum of biliary disease, as portrayed in Figure 5. Upon closer inspection of the figures, it was noteworthy that the US had a high detection rate of biliary disease, especially in the highest recurring diagnosis of acute cholecystitis (82 cases), followed by biliary colic (16 cases) and biliary pancreatitis (17 cases). 

Out of the 163 cases studied (139 individuals), 51 patients underwent cholecystectomy in our facility. The role of US was showcased by determining the sensitivity and specificity of its diagnostic strength when contrasted against patients who underwent surgical intervention in the form of laparoscopic cholecystectomy with histological assessment confirming the presence of stones. On follow-up review of the studied population, ultrasound showed a sensitivity of 48.72% and a specificity of 100% in the detection of gallstones. The reduced sensitivity value could be attributed to multiple factors, which include the passage of stones or the removal of stones in the operating theater to present to the patient prior to delivering the samples to histopathology.

Similarly, CT scans showed diverse results described in different articles in terms of the sensitivity and specificity of this modality, with values of sensitivity ranging between 50% and 100% and specificities ranging from 33% to 100% [16-18]. Regardless of this disparity, CT scans complement US in the clinical diagnosis process, most noticeable when findings are equivocal. Its role could be further appreciated in the stratification of severity in cases of associated pancreatitis, emphysematous cholecystitis, and potential perforation [14]. Our study has documented a sensitivity of 57.14% and a specificity of 100% when correlated with histological findings of gallstones following laparoscopic cholecystectomy performed in our facility. Furthermore, it was noted that the highest recurrence of diagnoses recorded were cases of acute cholecystitis (24 cases) and biliary pancreatitis (eight cases) in the diagnostic-positive category (Figure 7).

Despite being a benchmark in radiological assessment, US and CT scans have their own setbacks in the detection of certain biliary-related diseases and may provide inconclusive information. For instance, irrespective of its high sensitivity and specificity, US remains operator-dependent and does not provide 3-dimensional information, accurate measurements, or pathology characteristics, as evident by the lack of ability to confidently differentiate gallbladder polyps, gallstones, or main biliary tree disease. It is also dependent on the expertise of the radiologist and patient factors, such as obesity. On the other hand, a CT scan may fill in for ultrasound’s deficiency; however, it is restricted by its lesser sensitivity in the detection of biliary stone presence. This has led to the application and use of MR-based modalities for further assessment. Taking into consideration its limitations, including claustrophobia, overall cost, and contraindications to certain implanted devices and metallic objects, as well as the expertise required for image interpretation [19], it still remains a cornerstone for rapid and non-invasive assessment of biliary tree evaluation and diagnosis, where US and CT scans are found to be lacking, especially in detecting cholelithiasis, biliary obstruction, acute cholecystitis, biliary pancreatitis, malignancy, and patients who suffered traumatic injuries. Its utilization in an emergency setting varies in practice and is governed by the clinical scenario and availability [20].

Our study identified five cases that underwent MRCP/MRI; all of them featured diagnostic-positive results (Table 5). The distribution of the diagnoses can be found in Figure 8. This corresponded with the acknowledged worldwide role of MRCP/MRI.

Taking into consideration the prevalence of biliary disease, as extensively discussed throughout this study, it is not surprising that the private sector is heavily invested in its diagnostic process. Ultrasound is a considerably easy-to-use, safe, and relatively cheap device with reduced maintenance properties, making it desirable for private practice as a profit-generating commodity [21]. As such, 18 cases of admission in our sample study provided a history of radiological investigations done in external facilities. Of those, 14 cases underwent repeat investigations (Figure 9), for example, to rule out complications of gallstones and to evaluate the presence of perforation. A common theme for repeated imaging also included patients who failed to improve on conservative management, had a change in clinical picture, or had abnormal lab investigations, namely, deranged liver enzymes. Three cases had no clear indication for the repeated ultrasound; both local and private US reports showed identical findings, which could be argued due to distrust in the sonographic yield from another facility or other unmentioned reasons.

Furthermore, four patients with external US resorts proceeded to a repeated US in our facility and subsequently progressed to a delayed CT scan during their hospital course. These cases warranted advanced imaging to explore possible complications or the development of concomitant disease processes such as biliary pancreatitis secondary to cholecystitis and passed stones. This was driven by clinical suspicion and changes in lab investigations, such as high amylase levels or persistently deranged liver function tests.

The overall disease impact and hospital burden take into account various factors. For instance, the demographics of our data revealed that 38.7% of the study sample was composed of non-national (expats) males from the working-class age group and can be viewed as an extra governmental hospital burden and expense.

Another factor adding to the burden was the number of radiological requests being made and their overall contribution to the diagnosis process. Plain radiographs have an almost obsolete diagnostic value in regard to biliary disease; however, their use in excluding more serious and life-threatening conditions may prove essential when coupled with sensible clinical suspicion and assessment. Moreover, repeat imaging for patients with recent US reports available from an external source should be approached with caution and a low threshold for repeat imaging or proceeding to another radiological modality when in doubt.

**Figure 13:**
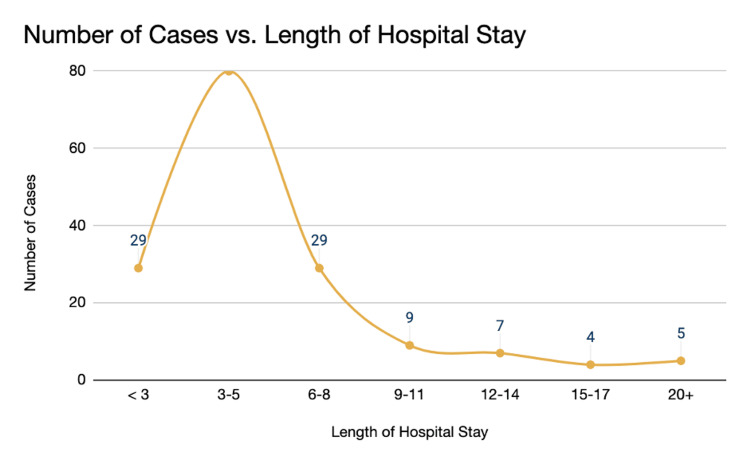
Length of hospital stay Number of cases (n=163) vs. the length of hospital stay in days (< 3 days to >20 days) < 3 days (n=29, 17.8%), 3-5 days (n=80, 49%), 6-8 days (n=29, 17.8%), 9-11 days (n=9, 5.5%), 12-14 days (n=7, 4.3%), 15-17 days (n=4, 2.5%), 20+ days (n=5, 3.1%)

The hospital stay also plays a major role in the accounting burden. Our data suggests that 29 cases had an admission period of (<3 days), with the majority of patients with biliary disease admitted for 3-5 days (n=80). Another 29 cases had a hospital course spanning 6-8 days. This has been in concordance with the average hospital stay in other countries [22] (Figure 13).

A complicated clinical outcome and prolonged hospital stay are directly correlated with increased hospital burden in terms of the extra expenses it incurs on the facility as well as the patient’s overall morbidity and mortality. This could be attributed to the increased workload of hospital staff members, prolonged bed occupancy, possible critical care admission, treatment administered, and repeated investigations (laboratory and imaging), in addition to the potential interventions needed, such as drainage procedures or even surgery.

As previously mentioned, the analyses of imaging modalities computed a single form of advanced radiological technique, excluding repeated requests from the same category or those images that were done beyond seven days from the index admission. As a result, subsequent imaging was done for further assessment of disease progression, evaluation of newly developed complications, or ruling out pathologies such as pulmonary emboli. However, these investigations and associated implications are without a doubt a taxing burden that adds to the overall economic toll exerted on medical facilities.

Readmission and emergency revisit rates go hand in hand with cumulative hospital service load; our study has estimated a 28% and 47.2% rate, respectively, with revisits accounting for at least one extra presentation to the emergency other than the index episode. One study estimated that around 13% of inpatients utilize more than half the hospital resources through repeated admissions [3]. Moreover, though the majority of gallstones may remain asymptomatic and be managed conservatively, there is a 2% yearly risk of progression to a symptomatic state [20].

On long-term follow-up of the patients who underwent laparoscopic cholecystectomy, two cases were done before their index episode and were admitted with surgical complications. 90% of our cases were operated on within nine months; two cases had the surgery in 18 months, and one case was done after 4.6 years. This lag between index admission and operation predisposes patients to higher rates of active disease. 

This study stems from primary research that looked into surgical emergency admission trends in the year 2018 to assess disease patterns and overall hospital load in our facility [23,24]. The process of calculating disease-related hospital burden can be appreciated by comparing the average length of hospital stay against the expected costs of overall medical services provided, in addition to recurrent disease and admission, which can complicate the hospital course. This topic remains beyond the scope of this study and will be explored in a future study currently underway.

A limitation to our study was noted in the acquisition of morbidity factors, which was due to a deficiency in documentation, namely obesity. As such, it might be susceptible to errors in documentation and clinical bias. As for the US and CT scan interpretation and results, those could have been affected by the limitations of the study in terms of sample size and operator dependency for the US, in addition to the duration between admission and the date of cholecystectomy. Furthermore, crude ultrasound sensitivity, as represented in Table 6, supports the diagnostic yield of ultrasound as the gold standard and most sensitive tool utilized. However, when contrasted against histopathology, a lower sensitivity yield of 48.72% was noted, which could be attributed to several factors. Most noteworthy are the passage of stones over time or stone extraction in the operating theater before submission of the specimen to the histopathology lab, and, hence, the specimen is reported without the presence of stones. Another limitation was in regards to the number of patients who did, in fact, undergo a laparoscopic cholecystectomy, as some cases might have already done the procedure in another facility or even abroad and were not accounted for.

## Conclusions

Biliary disease is a commonly encountered digestive disorder in the surgical field. Its prevalence and diverse spectrum necessitate the utilization of imaging modalities to assess, diagnose, and manage according to specific pathology processes.

Ultrasound is the preferred method and the gold-standard tool of choice, given its high detection rate, ease of use, availability, and relatively reasonable price compared to other modalities. In contrast, plain radiographs have no significant value in the detection of gallbladder-related disease, and their utilization should be limited when indicated rather than as a screening tool. Readmission rates have the highest effect on overall hospital burden, and early intervention yields more favorable outcomes as well as decreased resources spent. 

## Appendices

**Table 8:** Terminology Index

Terminology	Definitions	Value (n)
Total Number Of Surgical ER Admissions	Total number of hospital surgical admissions of all diseases between the 1st of January to 31st of December 2018 (n=1,546) + 16 episodes that were missing and added manually	1,562
Total Sample Size (Cases / Hospital Episodes)	All patients diagnosed with biliary tree disease in 2018 out of the total 1,562 surgical admissions in that year, this includes their readmission episodes	163
Number Of Patients / Individuals	The number of patients out of the 163 sample size adjusted after excluding their readmission episodes	139
Hospital Readmission	Number of patients (n = 139) who presented to the emergency department and were readmitted more than once in 2018 (i.e. >2 admissions)	21
Hospital Revisits	Number of cases (n=163) who visited the emergency department and were either discharged or admitted more than once within a 1-year time period (6 months before and 6 months) from the index admission	77
Nationals	Account of Bahrainis and General of the Gulf Cooperation Council (GCC) citizens	100
Non-Nationals (Expats)	Include patients without Bahraini nationality or not part of the GCC	63
Length Of Hospital Stay	Period extending from Date of Admission until Date of Discharge	N/A
Radiological Burden	Accounts for cumulative staff requirements, time invested as well as technology, general operation and maintenance cost.	N/A
Hospital Burden	Accounts for bed occupancy, cumulative staff requirements, administered medications and fluids, meals provided and overall nursing care in addition to radiological burden, laboratory investigations and surgical intervention	N/A
